# Induction of immunoglobulin G4 in human filariasis: an indicator of immunoregulation

**DOI:** 10.1179/136485910X12786389891407

**Published:** 2010-09

**Authors:** T. Adjobimey, A. Hoerauf

**Affiliations:** Institute for Medical Microbiology, Immunology and Parasitology, University Clinic Bonn, Sigmund Freud Strasse 25, 53105 Bonn, Germany

## Abstract

Filarial parasites are known to induce a large range of immunoregulatory mechanisms, including the induction of alternatively activated macrophages and regulatory T cells. These mechanisms are used to evade and down‐modulate the host’s immune system, to support parasite survival. Several reports have focused on some of these mechanisms, in humans and murine models, but the complex immunoregulatory networks associated with filarial infections remain unclear. Recent publications have conferred a role for regulatory T cells in the ability of helminth parasites to modulate human immune responses, such cells promoting the induction of the non‐complement‐fixing immunoglobulin G4 (IgG4). High plasma concentrations of IgG4 have been reported in hypo‐responsive and asymptomatic cases of helminth infection. In both human lymphatic filariasis and onchocerciasis, the asymptomatic infections are characterised by high plasma concentrations of IgG4 (compared with those of IgE) and of the complement‐fixing antibodies IgG1, IgG2 and IgG3. In asymptomatic filarial infection, elevations in IgG4 are also often associated with high worm loads and with high plasma levels of the immunomodulatory interleukin‐10. Here, various aspects of the induction of IgG4 in humans and it roles in the immunomodulation of the human responses to filarial parasites are reviewed.

Infections with filarial nematodes remain a major public‐health problem, especially in tropical countries (Kazura and Bockarie, 2003; Gbakima *et al*., 2005; Ramaiah *et al*., 2005; Njepuome *et al*., 2009). According to recent estimations (Molyneux, 2003; WHO, 2004), >200 million people are infected with the parasites that cause lymphatic filariasis (*Wuchereria bancrofti*, *Brugia malayi* and *B. timori*), onchocerciasis (*Onchocerca volvulus*) or loiasis (*Loa loa*). These infections are often chronic and persist for many years. In most infected individuals, however, few, if any, symptoms develop, the relatively asymptomatic existence of filarial parasites in the host being the result of the ability of filarial nematodes to down‐modulate the host’s immune responses, in order to facilitate their own survival. Unfortunately, some infected individuals do develop serious immunopathology. The immunological and genetical factors influencing the direction that any filarial infection takes, towards immunopathology or to tolerance, are still not fully understood.

Induction of the non‐cytolytic and blocking immunoglobulin G4 (IgG4) is believed to represent one major mechanism used by filarial parasites to evade destruction by their host’s immune system. The immunoregulatory properties of IgG4 have been recently demonstrated (Van der Neut Kolfschoten *et al*., 2007). This immunoglobulin seems to have markedly different properties to those of other immunoglobulins of the G subclass. Although the blocking effects of IgG4 in allergic diseases are well known (Taylor *et al*., 2004; Akdis *et al*., 2005, 2006), the function of this immunoglobulin in filarial infections is not well documented. Some aspects of the immunoregulatory role of IgG4 in the pathology of three major filarial infections (lymphatic filariasis, onchocerciasis and loiasis) are reviewed below.

## DISEASE AND THE SUBCLASSES OF IMMUNOGLOBULIN G

The four subclasses of IgG differ in both their physical and biological properties. It is, for example, well known that IgG1 antibody responses are prominent in humans with microbial infection. Although such responses are usually driven by Th1 cells, IgG1 is not specific to Th1. In fact, IgG1 responses more often correlate with IgE expression, which is mainly related to Th2. Unlike the situation in mice models, where clear Th1/Th2 polarizations can be observed, both Th1 and Th2 cytokines have been shown to be able to drive IgG1 in humans (Mosmann and Coffman, 1989; Kotowicz and Callard, 1993; Fujieda *et al*., 1995; Fear *et al*., 2004). In various experimental models, helminth antigens and allergens have been shown to induce IgG4 and IgE, both *in vitro* and *in vivo* (Lobos *et al*., 2003), whereas other parasite antigens, such as those of malarial parasites, induce IgG1 and IgG3 (Garraud *et al*., 1995, 2002, 2003*a*). These differences are often controlled by the cytokines that the antigens induce. Although naïve B cells produce IgG4 and IgE in the presence of interleukin‐4 (IL‐4) and interleukin‐13 (IL‐13), they produce IgG2 in the presence of interferon‐γ (IFN‐γ) and interleukin‐12 (IL‐12; Garraud *et al*., 2003*b*). In mice, interleukin‐21 (IL‐21) appears to have a critical role in delineating between the production of IgG1, the likely equivalent of human IgG4, and IgE (Ozaki *et al*., 2002; Aalberse *et al*., 2009). Similarly, IL‐21 has been implicated in the preferential secretion of IgG4 by human leucocytes (unfractionated peripheral‐blood mononuclear cells) that have been stimulated with phytohaemagglutinin (Wood *et al*., 2004). These findings, together with the fact that IL‐21 can be secreted by Th17 cells, indicate a possible role for Th17 cells in the regulation of the IgG subclasses (Wei *et al*., 2007; Jang *et al*., 2009; Liu *et al*., 2009). The possible modulation of B‐cell responses by Th17 cytokines remains to be studied. Interleukin‐10 (IL‐10), tumour growth factor‐β (TGF‐β) and other immunoregulatory cytokines are also often implicated in IgG4 production in humans (Satoguina *et al*., 2002; see Table).

**Table:** Immunoglobulins and their corresponding stimuli and T‐cell responses

Immunoglobulin	Cytokines	Antigens	Corresponding T‐cell response
IgG1	(Interferon‐γ, interleukin‐12)	Bacteria, viruses	Th1 and Th2
IgG2	Interferon‐γ, interleukin‐12		Th2 and Th1
IgG3	–	–	Th2 and Th1
IgG4	Interleukin‐4, ‐10, ‐13 and ‐21	Large parasites, allergens	Th2, regulatory T cells
IgE	Interleukin‐4 and ‐13	Large parasites, allergens	Th2
IgA	–	–	
IgM	–	–	

## IMMUNOREGULATORY PROPERTIES OF IMMUNOGLOBULIN G4

IgG represents one of the most important classes of immunoglobulin, molecules of this class being secreted relatively late in an immune response, after the B cells have gone through affinity maturation, allowing a greater affinity of antibodies during persistent antigen exposure. IgG molecules are also secreted by memory B cells during secondary immune responses. Although all IgG molecules have the same overall structure, there are many points of difference between IgG4 and the other members of the IgG class (Burton *et al*., 1986; Simpson *et al*., 1990; Yoshida *et al*., 2006; Petrie and Zúñiga‐Pflücker, 2007; Wang *et al*., 2008) and these differences are not only structural but also functional (Burton *et al*., 1986; Simpson *et al*., 1990). While IgG1, IgG2 and IgG3 are able to fix and activate complement, IgG4 has no affinity for complement and is therefore unable to activate any of the protective immune mechanisms that involve complement. Furthermore, in contrast to IgG1 and IgG3, IgG4 cannot induce antibody‐dependent cell‐mediated cytotoxicity (ADCC) after binding to the FcγRIII molecule, CD16, found on the surface of neutrophils and eosinophils (Dafa’alla *et al*., 1992). IgG4 is also known to compete with IgE for the antibody fixation sites on mast cells and eosinophils (Zola *et al*., 1978; Aalberse *et al*., 2009). While IgE induces such cells to degranulate, IgG4 has no or few downstream effects and can even inhibit complement activation by other antibodies, as demonstrated in a phospholipase‐A model (Van der Zee *et al*., 1986; Aalberse *et al*., 2009). Another property of IgG4 was described only a few years ago. A day after injecting nude mice with blood‐derived or recombinant IgG4 antibodies against the major birch‐pollen antigen (Betv1) and with similar antibodies against a cat allergen (Feld1), Van der Neut Kolfschoten *et al*. (2007) demonstrated the development of IgG4 molecules with double specificity (i.e. to both Betv1 and Feld1). This observation indicated that an IgG4 molecule is a dynamic molecule that can swap a heavy chain and attached light chain (half‐molecule) for a heavy–light chain‐pair from another molecule (Van der Neut Kolfschoten *et al*., 2007; Aalberse *et al*., 2009). Such an exchange of Fab arms is probably responsible for some of the immunoregulatory properties of IgG4 seen in a rhesus‐monkey model of auto‐immune myasthenia gravis — a disease in which auto‐antibodies against an acetylcholine receptor (AChR) induce muscle weakness (Losen *et al*., 2005). By injecting rhesus monkeys with a human AChR‐specific antibody (IgG1‐637 — a patient‐derived antibody that induces AChR degradation), auto‐immune myasthenia gravis can be induced (Graus *et al*., 1997). Monkeys co‐injected with IgG4‐637, however, remain healthy, indicating a protective role for IgG4 in at least one inflammatory disease (Losen *et al*., 2005). A dynamic arm‐exchange mechanism might play a crucial role in the protective role of IgG4 again lymphoedema in lymphatic filariasis and allergic diseases (Hussain and Ottesen, 1986).

## CORRELATION OF IGG4 LEVELS WITH THE ABSENCE OF PATHOLOGY IN HUMAN LYMPHATIC FILARIASIS

Human lymphatic filariasis, also known as elephantiasis, is caused by three species of filarial parasites: *W. bancrofti*, *B. malayi* and *B. timori*. While most people who are infected with one of these parasites remain asymptomatic, some develop visible manifestations such as lymphoedema of the limbs and/or the swelling of the scrotum and penis known as hydrocele (Pfarr *et al.*, 2009). Infection with any of these nematodes is associated with a strong polarization of the host’s immune response towards Th2, with prominent secretion of IL‐4 and IL‐5 and elevated levels of IgG4 and IgE (Hussain *et al.*, 1981; Ottesen *et al.*, 1985; Hussain and Ottesen, 1986; Mahanty *et al.*, 1993). Ottesen *et al*. (1985), for example, observed an extreme elevation of both total and, particularly, filarial‐antigen‐specific IgG4 in humans infected with *W. bancrofti*. Such elevations in IgG4 indicate the importance of this immunoglobulin in the human immune response to filarial infection (Noordin *et al*., 2003; Jiraamonnimit *et al*., 2009); most (50%–95%) of the antifilarial IgG in cases of *W. bancrofti* filariasis belonging to this subclass (Ottesen *et al*., 1985). Several experimental observations indicate that it is the parasites’ microfilariae that are the main inducers of IgG4. Shiny *et al*. (2009) recently reported a 54‐year‐old man from Haiti who, although initially microfilaraemic (with 100 microfilariae/ml blood) and seropositive for antifilarial IgG4, became amicrofilaraemic (and persistently seronegative for antifilarial IgG4) after treatment with diethylcarbamazine but developed lymphoedema in one leg approximately 1 year later. In this patient, titres of antifilarial IgG1 began to increase after the development of the lymphoedema, peaking after a further 2 years (Shiny *et al*., 2009). Similar observations were made by Kurniawan *et al*. (1993) while comparing antibody secretion in individuals resident in an area of Indonesia that was endemic for lymphatic filariasis caused by *B. malayi*. In this investigation, antibody levels to antigens extracted from adult *B. malayi* were determined for each of the IgG subclasses as well as for IgM and for IgE. The predominant isotype of antifilarial antibody was found to be IgG4, which, in asymptomatic microfilaraemics, represented 88% of the total IgG. Interestingly, the patients in this Indonesian study who had chronic disease (elephantiasis) were generally amicrofilaraemic and had substantially higher levels of IgG1, IgG2 and IgG3 but, on average, 3.4‐fold lower levels of specific IgG4 than the asymptomatic microfilaraemics. Kurniawan *et al*. (1993) also found that, in contrast to the trends in IgG4, the levels of antifilarial IgE were, on average, 4.5‐fold higher in the cases of elephantiasis than in the asymptomatic carriers. Taken together, these observations clearly demonstrated a protective role for IgG4 that seems to inhibit the development of elephantiasis. In their investigations of sera from patients with *W. bancrofti* filariasis, Hussain *et al*. (1992) found that, among the four IgG subclasses, only the levels of IgG4 correlated with the blocking activity observed in histamine‐release assays. In addition, the blocking activity detected in the sera showing high levels of histamine inhibition could be abolished by the selective depletion of the IgG4 in such sera, using anti‐IgG4 affinity columns (Hussain *et al*., 1992).

## ROLE OF IGG4 IN THE PATHOLOGY OF HUMAN ONCHOCERCIASIS

The causative agent of human onchocerciasis is the filarial nematode *O. volvulus*. The adult parasites reside mainly in subcutaneous nodules (Brattig, 2004). The adult females produce thousands of microfilariae that migrate in the skin and to other tissues, causing various pathological manifestations (Korten *et al*., 2010*a*). Migration of the microfilariae into the eyes can cause blindness, the most severe manifestation of the disease. Most infected individuals are hypo‐responsive (in the generalized form of the disease) and present with high loads of microfilariae and adult worms. In contrast, a small number of infected individuals present with low parasitaemia but severe and chronic dermatitis (in the hyper‐reactive form of the disease, sometimes known as sowda). Untreated cases of sowda are usually able to eliminate their microfilaraemias by mounting a strong, local and systemic Th2 immune response (Brattig *et al*., 2002; Hoerauf and Brattig, 2002; Brattig, 2004) but this strong immune reaction is often associated with severe immunopathology. The humoral response in such cases is dominated by the production of both antigen‐specific and total IgE, IgG1 and IgG3. High IgG4 production is, however, the hallmark of the other, hypo‐reactive cases.

More IgG4‐positive plasma cells are detectable in onchocercomas from the hypo‐reactive cases of onchocerciasis than in those from hyper‐reactive patients (Brattig *et al*., 2010; Korten *et al*., 2010*a*). The results of a study by Dafa’alla *et al*. (1992) illustrated the prominence of IgG4 in human onchocerciasis and the role of such immunoglobulin in the survival of microfilariae. Dafa’alla *et al*. (1992) compared the IgG responses to *O. gutturosa* and *O. volvulus* and concluded that IgG4 secretion, in response to *O. volvulus* or *O. gutturosa*, was considerably higher than IgG1, IgG2 or IgG3 secretion (the ELISA used giving mean ‘corrected’ optical densities of 0.84, 0.27, 0.24 and 0.28, respectively). They also demonstrated that IgG4 levels were positively correlated with microfilaraemia, whereas IgG3 levels showed a negative association with the microfilarial load. The high levels of IgG4 antibody in individuals with high microfilarial loads indicate a role for this isotype in inhibiting microfilarial clearance. Dafa’alla *et al*. (1992) suggested that IgG4 competed with other antibodies, probably IgE and IgG3, for adherence to the microfilariae and thereby inhibited the ADCC known to be involved in microfilarial destruction (Subrahmanyam *et al*., 1978; Haque *et al*., 1981).

## IGG4 IN HUMAN LOIASIS

Loiasis is a skin and eye disease caused by *Loa loa* filarial worms. The adult worms produce microfilariae that can be found in blood and other body fluids and in the lung (Agbolade and Akinboye, 2001; Padgett and Jacobsen, 2008). The main clinical sign is the ‘Calabar swelling’, which is oedema in the subcutaneous tissue caused by maturing larvae migrating away from the site where they were injected by a feeding vector fly. Migration of the worms through the eye causes severe eye pain, inflammation and sometimes blindness (Boussinesq, 2006). In Central and West Africa, individuals with high loads of *L. loa* microfilariae are at risk of developing serious neurological reactions after treatment with the diethylcarbamazine or ivermectin used in mass treatments for the elimination of onchocerciasis (Pion *et al*., 2005). Loiasis differs from the other filarial diseases of humans in that most infected subjects are without circulating microfilariae (i.e. they have ‘occult’ loiasis). Most cases of loiasis are also asymptomatic (Agbolade and Akinboye, 2001; Padgett and Jacobsen, 2008).

In a study in two villages in south–eastern Gabon (one with high‐intensity transmission of *L. loa* and one with low‐intensity transmission), Akue *et al*. (2002) found that plasma concentrations of microfilaria‐ and adult‐worm‐specific IgG4 were significantly higher in the microfilaraemics than in the amicrofilaraemic villagers. In contrast, levels of IgG1 specific to the third‐stage larvae or microfilariae of *L. loa* were significantly higher in the amicrofilaraemic subjects than in the microfilaraemic. These observations indicate that *L. loa* microfilariae are at least partially responsible for the preferential production of IgG4 in human loiasis. The absence of microfilariae is often associated with the production of the more immunocompetent immunoglobulins IgG1 and IgE, which often appear associated with the development of immunopathology. Curiously, in an earlier study in Gabon by the same research group, similarly high levels of IgG4 expression were found in subjects with and without *L. loa* microfilaraemias (Akue *et al*., 1994) and it seems possible that transmission intensity has a major effect on the antibody responses to *L. loa* infection (Akue *et al*., 1994, 2002).

In conclusion, it is likely that, in general, the presence of *L. loa* microfilariae actively down‐regulates IgG1 levels while inducing IgG4, changes which, in turn, promote the survival of the microfilariae and adult worms.

## CELLULAR MECHANISMS OF PREFERENTIAL IGG4 INDUCTION IN FILARIASIS

The mechanisms used by filarial parasites to suppress a host’s immune responses are diverse and multiform. Although the preferential induction of IgG4 is one important arm of this immunoregulatory network, the mechanisms that lead to IgG4 production are still not fully characterised. It is known that microfilariae can induce two immunoregulatory cytokines (TGF‐β and IL‐10) as well as IL‐10‐producing and CD4(+)CD25(+)FOXP3(+) regulatory T cells (Taylor *et al*., 2009; Korten *et al*., 2010*b*; Metenou *et al*., 2010). Since regulatory T cells and IL‐10 are known to promote the production of IgG4 by B cells (Satoguina *et al*., 2005, 2008), microfilariae may well be directly responsible for IgG4 production. The induction of IgG4 by regulatory T cells has also been observed in allergy models. Meiler *et al*. (2008), for example, demonstrated that circulating CD4(+)CD25(+)FOXP3(+)regulatory T cells and allergen‐specific, IL‐10‐secreting, type‐1 regulatory T cells from healthy individuals were able to induce IgG4, while suppressing IgE production in peripheral‐blood mononuclear cells and purified B‐cell cultures.

In their recent study, Satoguina *et al*. (2008) showed that both soluble factors and cell‐contact‐dependent mechanisms are involved in the induction of IgG4 by regulatory T cells. By using clones of human IL‐10‐producing, antigen‐specific regulatory T cells, in co‐culture with autologous B cells, it was demonstrated that the blocking of GITR (glucocorticoid‐induced tumour‐necrosis‐factor receptor‐related protein) or GITR ligand (GITR‐L) selectively prevented IgG4 production, as did the inhibition of IL‐10 or TGF‐β. In addition, the prevention of IgG4 induction with an anti‐GITR antibody could be reversed using an excess of recombinant IL‐10 but not by using recombinant TGF‐β (Satoguina *et al*., 2008). These observations indicated that IL‐10 plays the terminal role in a cascade of signals, including GITR, GITR‐L and TGF‐β, that leads to the production of IgG4.

It may be the IgG4/IgE or IgG4/IgG ratio that is one of the main criteria determining whether a filarial infection heads toward immunoregulation (and an asymptomatic state) or immunopathology. Hence, IL‐10 from microfilariae‐induced regulatory T cells might, in the context of an immunological synapse, induce B cells to secrete IgG4 preferentially, promoting parasite tolerance. Toll‐like receptors that are known to be expressed on regulatory T cells (Nyirenda *et al*., 2009; Urry *et al*., 2009) might be able to modulate the IgG4 induction.

## CONCLUDING REMARKS

Filarial parasites are responsible for peripheral‐T‐cell tolerance, at least partially through the induction of IL‐10‐producing regulatory T cells and the recruitment and expansion of naturally occurring regulatory T cells. Natural CD4(+)CD25(+)FOXP3(+) regulatory T cells and antigen‐induced, IL‐10‐producing, type‐1 regulatory T cells modulate the host’s immune responses by enhancing the production of non‐cytolytic IgG4 antibodies. The IgG4 molecules are capable of inhibiting IgE‐ and IgG‐mediated effector mechanisms. This humoral regulation makes an important contribution to the avoidance of pathology (e.g. filarial lymphoedema, onchocercal dermatitis, keratitis and/or tropical pulmonary eosinophilia). In the absence of immunoregulation, the host’s immunocompetent antigen‐presenting cells activate effector T‐cells, which, in turn, induce B cells to produce cytolytic antibodies (IgG1, IgG2, IgG3 and IgE). These antibodies, through different effector mechanisms (e.g. complement activation and ADCC), induce parasite death and the subsequent release of antigens from endosymbobiotic *Wolbachia* bacteria. These bacterial antigens contribute to the induction of a strong immune reaction and, subsequently, to the development of pathology (see Figure). A better understanding of the genetic and immunological factors that induce the immunoregulatory mechanisms seen in human filariasis would surely contribute to the design of more efficient and safe therapies against filarial infections.

**FIG:**
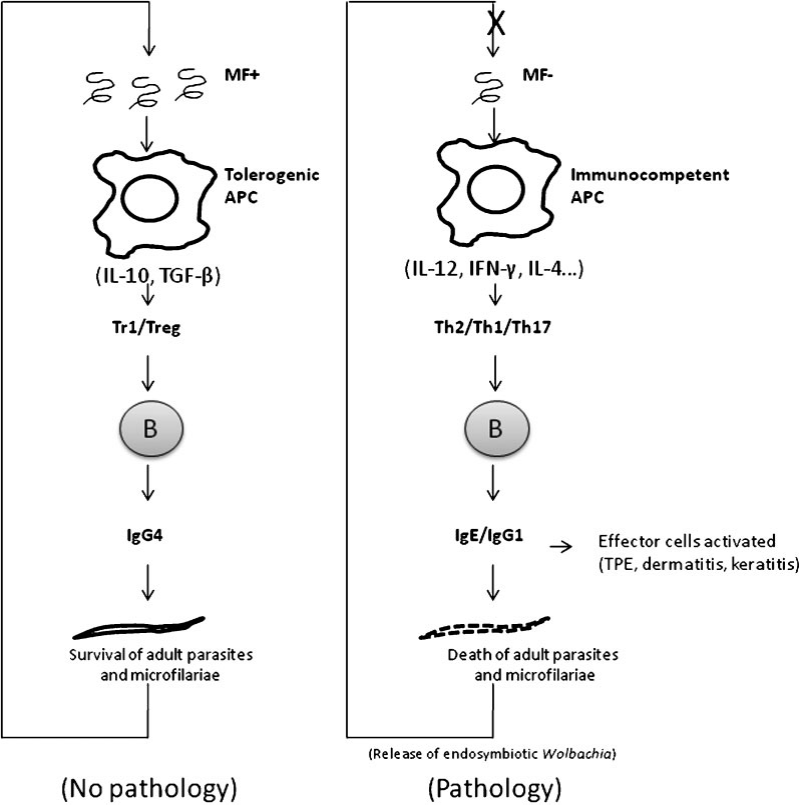
Simplified view of the induction and regulatory properties of IgG4 in human filariasis. Adult filarial parasites produce microfilariae (MF) that are responsible for the recruitment and induction of Foxp3(+) and interleukin‐10‐producing regulatory T cells (Treg), probably by the manipulation of antigen‐presenting cells (APC). Natural CD4(+)CD25(+)FOXP3(+) Treg and antigen‐induced, interleukin‐10‐producing, regulatory cells of type 1 (Tr1) interact with B cells and enhance the production of non‐cytolytic IgG4 while inhibiting the induction of other IgG and IgE. This humoral regulation contributes to the avoidance of pathology [e.g. filarial lymphoedema, onchocercal dermatitis, keratitis and tropical pulmonary eosinophilia (TPE)]. In the absence of immunoregulation, immunocompetent APC activate effector T‐cells (Th) which, in turn, induce B cells to produce cytolytic IgG1, IgG2, IgG3 and IgE. These antibodies induce various effector mechanisms (such as complement activation and antibody‐dependent cell‐mediated cytotoxicity), provoking parasite death and the release of antigens from endosymbobiotic *Wolbachia*. These bacterial antigens contribute to the induction of a strong immune reaction and to the subsequent development of pathology. IL‐10, Interleukin‐10; TGF‐β, tumour growth factor‐β; IL‐12, interleukin‐12; IFN‐γ, interferon‐γ; IL‐4, interleukin‐4.
